# Real-Time Fluoroscopic and C-Arm Computed Tomography Evaluation of Ommaya Reservoir Integrity

**DOI:** 10.7759/cureus.1097

**Published:** 2017-03-15

**Authors:** Adrienne M Moraff, Melanie Hayden Gephart, Larry M Shuer, Jeremy J Heit

**Affiliations:** 1 Department of Neurosurgery, Stanford University School of Medicine; 2 Stanford University School of Medicine; 3 Department of Radiology, Stanford University School of Medicine

**Keywords:** ommaya reservoir, c-arm ct, leakage, tubing, subdural hematoma, flat panel ct

## Abstract

We describe a case of a 24-year-old patient with relapsed acute myelogenous leukemia involving the central nervous system. After placement of an Ommaya reservoir for intrathecal chemotherapy administration, the patient developed progressive headache, nausea, and drowsiness and was found to have an enlarging subdural collection underlying the Ommaya. To exclude leakage of the Ommaya system into the subdural space, real-time fluoroscopic and C-arm computed tomographic evaluation of the Ommaya reservoir was performed after iodinated contrast injection into the reservoir. This novel technique demonstrated complete integrity of the Ommaya reservoir without evidence of blockage or leakage of the system. The patient underwent uncomplicated evacuation of the subdural collection without replacement of the Ommaya reservoir and made an excellent recovery. This technique for real-time interrogation of the Ommaya reservoir may have additional utility in the evaluation for Ommaya reservoir dysfunction.

## Introduction

Ommaya reservoirs are surgically implanted closed catheter systems with a needle-accessible subcutaneous reservoir [1]. This reservoir is in continuity with the cerebrospinal fluid (CSF) through tubing that extends from the reservoir to the ventricular system, which allows for repeated CSF sampling and intrathecal medication delivery [2]. Ommaya implantation is generally well tolerated, but complications related to the device implantation occur in 15% of patients [3-4]. Infection, intraparenchymal or intraventricular hemorrhage, catheter malposition, CSF leakage, tubing blockage, and development of a subdural hematoma or hygroma are among the most commonly encountered complications, and these complications may require surgical revision [4-5].

Neuroimaging is performed to evaluate Ommaya reservoir placement and to assess for complications related to placement. Most commonly computed tomography (CT) or magnetic resonance imaging (MRI) is performed to determine the location of the Ommaya catheter tip, to assess for intracranial hemorrhage, and to identify extra cerebral collections following placement. Although CT and MRI offer excellent spatial resolution and information regarding the adjacent brain parenchyma and ventricular system, these techniques do not evaluate the dynamic flow of fluid through the Ommaya reservoir, which limits its ability to detect tubing blockage or the site of leakage.

Shunt interrogation studies (termed “shuntograms”) have also been used to assess the integrity of other ventriculoperitoneal shunts. In these studies, iodinated contrast is injected into the shunt tubing, and serial radiographs are taken every 15-20 minutes to evaluate for contrast extravasation along the course of the tubing rather than in the peritoneum, where the tubing terminates. This technique may also be adapted to Ommaya reservoirs, although such an adaptation has not been reported.

Radioisotopes, including 111In-DPTA or 99mTc-DPTA, may be introduced through an Ommaya to assess the CSF flow blockage, Ommaya tubing blockage, or Ommaya leakage by radionuclide imaging techniques [6-8]. However, radionuclide imaging has poor spatial and temporal resolution, which may prevent clear identification of the site of leakage or blockage [9]. To prevent unnecessary Ommaya replacement when compromise of the reservoir or tubing is incorrectly suspected, better minimally-invasive dynamic diagnostic testing of Ommaya system integrity is needed.

Here we describe a novel real-time imaging technique for evaluation of Ommaya reservoir integrity in a patient with a symptomatic post-operative subdural collection. The Ommaya reservoir was injected with iodinated contrast, and transit of this contrast through the Ommaya system into the ventricular system was well visualized with real-time fluoroscopy to exclude leakage of the Ommaya system into the subdural collection. A flat panel CT was obtained in the neuroendovascular suite for further evaluation of the subdural collection after contrast injection, and reformatted images from this study demonstrated no contrast extension into the subdural collection. Based on these imaging findings, the patient underwent uneventful evacuation of the subdural collection without replacement of the Ommaya shunt, and he made an excellent recovery.

## Case presentation

A 24-year-old man with a nine-month history of acute myelogenous leukemia (AML) presented to our hospital with progressive headache, nausea, and drowsiness. The patient initially presented 11 months earlier with fatigue, and he was admitted to an outside hospital. He was found to be anemic, with a complete cell count demonstrating evidence of AML with a hemoglobin level of 5.1 g/dl, a white blood cell count of 14.1 x 10^3^/µL with 89% blasts, and platelets of 29 x 10^3^/µL. A complete evaluation at the time of diagnosis demonstrated bone marrow infiltration, but no evidence of central nervous system involvement. The patient underwent induction chemotherapy treatment with high dose pravastatin, idarubicin, and cytarabine, and he required re-induction for a persistent blast level of 78% on a bone marrow aspiration that was performed 14 days after induction. Following re-induction therapy, a repeat bone marrow biopsy showed no blasts, and he underwent consolidation chemotherapy with high-dose cytarabine followed by a matched myeloablative stem cell transplantation.

**Figure 1:**
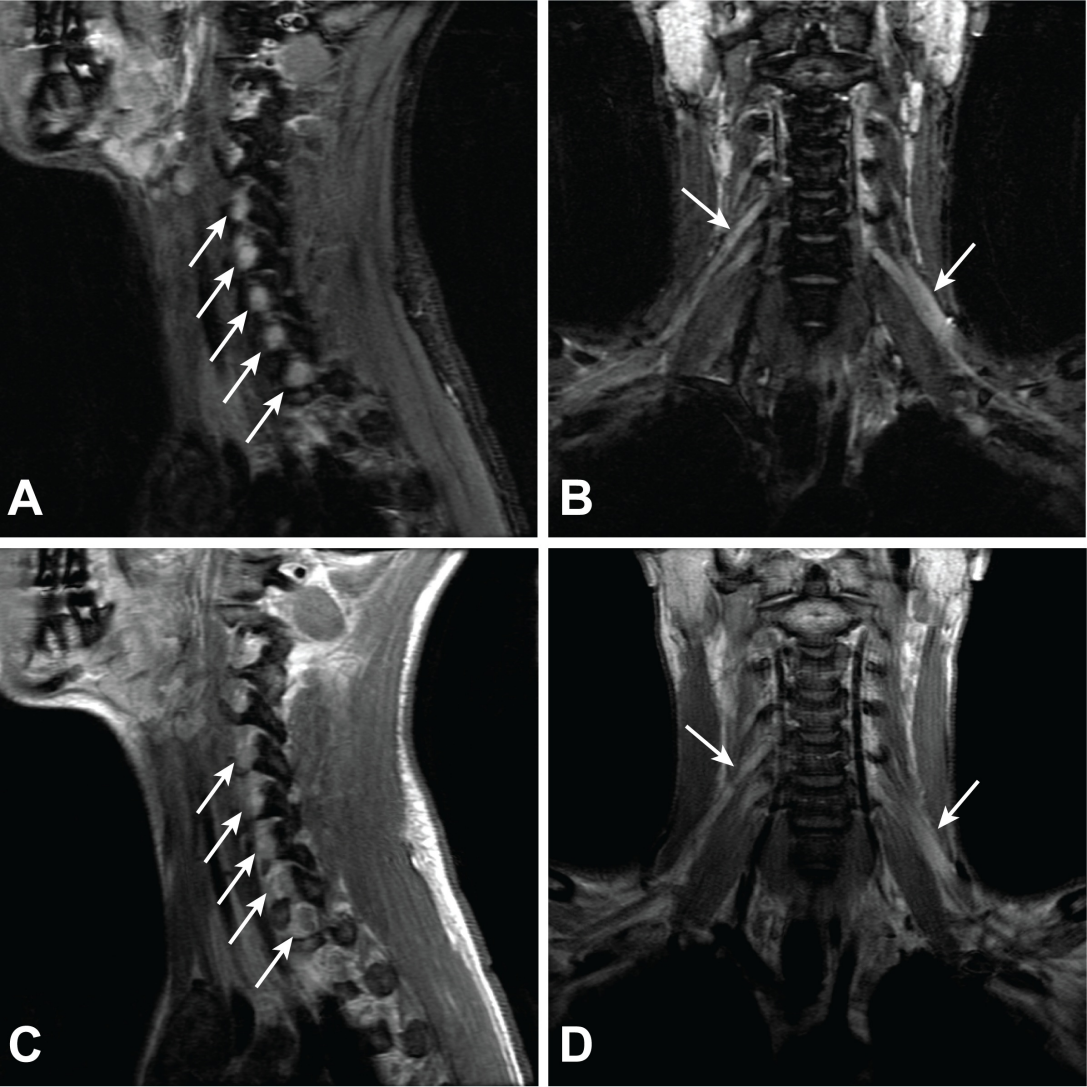
Brachial plexus magnetic resonance imaging demonstrating leukemic infiltration of the bilateral nerve roots. (A, B) Short tau inversion recovery (STIR) images demonstrate nerve root thickening and increased T2-weighted signal abnormality (arrows) in the sagittal (A) and coronal plane (B). (C, D) Post contrast images show diffuse enhancement within thickened nerve roots of the brachial plexus in the sagittal (C) and coronal plane (D).

The patient initially did well after his bone marrow transplant, but nine months later he developed neck spasms and right upper extremity neuropathy. He underwent an MRI scan of the right brachial plexus (Figure 1), which demonstrated nerve root thickening that was found to represent leukemic infiltration. A lumbar puncture demonstrated evidence of blasts in the CSF. There was no evidence of peripheral disease recurrence upon repeat bone marrow biopsy and a complete imaging evaluation. The patient underwent treatment with high-dose cytarabine and dexamethasone. A right frontal Ommaya reservoir was placed using stereotactic guidance for weekly intrathecal chemotherapy.

**Figure 2:**
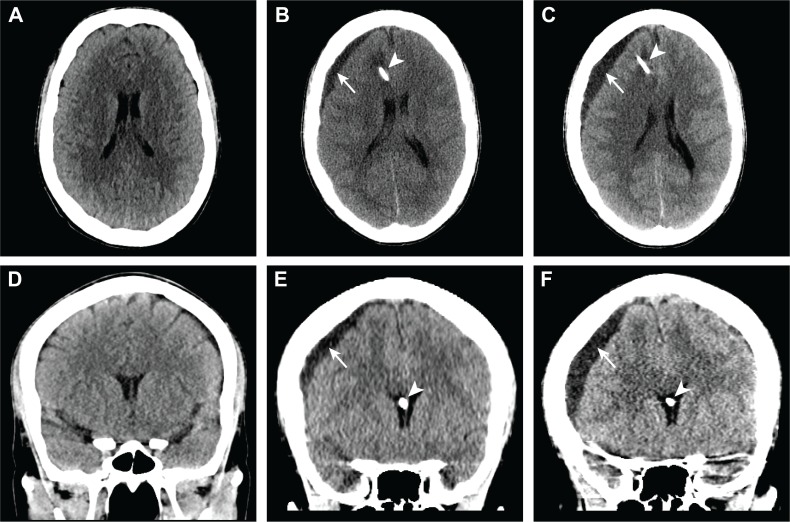
Head computed tomography images before and after Ommaya reservoir placement demonstrate increasing size of a right subdural collection. Axial (A-C) and coronal reformations (D-F) from a non-contrast head computed tomogram demonstrate a normal appearance of the brain prior to Ommaya reservoir placement (A, D). After Ommaya reservoir placement, the catheter (B, C, E, F, arrowheads) traverses the right frontal lobe (B, C) and terminates in the right lateral ventricle near the foramen of Monro (E, F). There is a hypodense subdural collection (B, C, E, R, arrows) overlying the right frontal lobe that was initially small (B, E) but increased on a follow-up study (C, F).

The patient developed a headache seven days after placement of the Ommaya reservoir, and there was difficulty in accessing the Ommaya. A head CT demonstrated appropriate positioning of the Ommaya reservoir tip in the frontal horn of the right lateral ventricle, but a new hypodense 5-mm extra-axial collection overlying the right frontal lobe was present (Figure 2). The patient’s headache worsened, and he developed nausea and vomiting with intrathecal chemotherapy administration. A follow-up head CT demonstrated an interval increase in the size of the right frontal extra-axial collection with an associated increase in local mass effect on the adjacent cerebral sulci and modest midline shift.

The patient’s symptoms persisted for an additional two weeks, and he developed increasing confusion and somnolence. The right frontal extra-axial collection remained stable in size, but the concern was raised that the Ommaya reservoir system had been damaged during needle access for intrathecal chemotherapy administration. It was further postulated that leakage of the Ommaya system into the subdural space overlying the right frontal lobe might account for the right frontal lobe extra-axial collection. Other considerations for the patient’s symptoms and imaging findings included brain shift and draining vein tension with fluid removal and administration of chemotherapy, medication leakage into the subdural space during chemotherapy administration, or intracranial hypotension given the patient’s history of prior lumbar punctures. The patient was planned for surgical drainage of the extra-cerebral collection and possible replacement of the Ommaya reservoir. Given his neutropenic status, it was preferred that the Ommaya only be removed and replaced if it was damaged.

**Figure 3:**
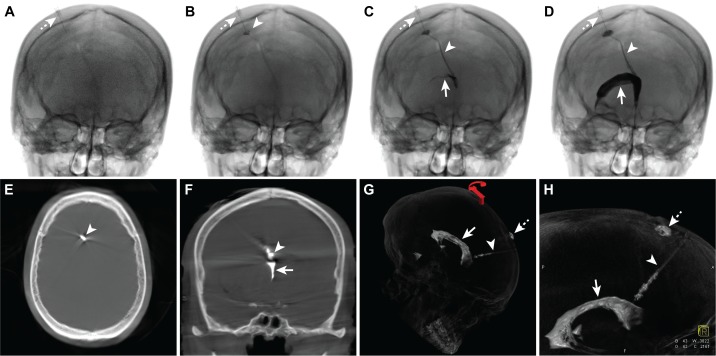
Fluoroscopic images during contrast injection of the Ommaya reservoir and flat panel computed tomography images after contrast injection. Serial fluoroscopic images (A-D) obtained during iodinated contrast injection of the Ommaya reservoir demonstrate the needle in position within the reservoir (A-D, dashed arrow) prior to injection (A) and during injection (B-D). There is progressive contrast opacification of the Ommaya reservoir (B, arrowhead), tubing (B, C, arrowhead), and right lateral ventricle (D, arrow), but no contrast extends into the subdural space. (E-H) Three-dimensional images reformatted from a flat panel computed tomography study after Ommaya reservoir contrast injection. There is no contrast evidence within the right frontal subdural collection (E, F). The Ommaya tubing is well seen with contrast within it (E, F, arrowhead), and contrast is noted filing the third ventricle on the coronal image (F, arrow). Additional reformatted images (G, H) demonstrate contrast filling the Ommaya reservoir (G, H, dashed arrow), tubing (G, H, arrowhead), and right lateral ventricle (G, H, arrow). No contrast extends outside of the Ommaya reservoir or ventricle system into the subdural space.

To assess the integrity of the Ommaya system and leakage into the subdural space, the patient was brought to the neuroendovascular suite for real time fluoroscopic evaluation of the Ommaya following contrast injection of the reservoir. The neuroendovascular suite is equipped with a Siemens Artis Zee biplane fluoroscopy unit with flat panel CT capability. The patient was placed supine on the angiography table. The skin overlying the Ommaya reservoir was sterilely prepared and draped in a standard fashion, and the reservoir was accessed with a 22-guage butterfly needle. Return of clear CSF was verified after this access, and 5 ml of Omnipaque 300 contrast dye was slowly injected into the Ommaya reservoir. The contrast injection was monitored with continuous low-dose single-plane fluoroscopy (Figure 3A-D). The injected contrast remained confined to the Ommaya reservoir and catheter, and there was an appropriate filling of the right lateral and third ventricles. There was no contrast leakage into the subcutaneous tissues, subdural space, or brain parenchyma. Similarly, no blockage or delayed flow of contrast was observed to suggest blockage within the Ommaya system. A flat panel CT was then performed, and three-dimensional reformations were made on a separate workstation (Figure 3E-H). These images again demonstrated no evidence of contrast leakage into the subdural space or other location outside the Ommaya reservoir or normal CSF space. The butterfly needle was removed. The patient tolerated the procedure well, and there were no complications related to the procedure.

The patient was transferred to the operating room, where a right frontal burr hole craniotomy posterior and separate from the Ommaya incision was performed. A hemosiderin-stained clear subdural collection was evacuated without difficulty, and the underlying brain expanded intra-operatively after this evacuation. The patient recovered uneventfully from the surgery, and his headache, nausea, and vomiting resolved. He was discharged home seven days after the evacuation of the subdural collection. 

## Discussion

Ommaya reservoirs are surgically implanted catheter systems that allow for repeated access to the CSF and intrathecal chemotherapy administration, which decreases patient morbidity related to repeated lumbar puncture [1-2]. However, Ommaya reservoirs may develop blockage within the catheter or leakage due to damage of the reservoir, damage to the catheter, or disconnection of the reservoir and catheter components [4-5]. Compromise of the Ommaya system may result in non-target delivery of chemotherapy or a CSF leak that may lead to the development of an extra-axial fluid collection. Surgical replacement of the Ommaya may be required in the setting of Ommaya reservoir compromise, and there is an increased risk of infection and bleeding complications in this patient population that is frequently neutropenic and thrombocytopenic.

Non-invasive evaluation of Ommaya reservoir integrity is challenging given the need for high temporal and spatial resolution when interrogating the system. Here we describe, to our knowledge, the first description of real-time fluoroscopic interrogation of Ommaya reservoir integrity. Fluoroscopy provides excellent temporal and spatial resolution, and iodinated contrast injection of the Ommaya reservoir allowed for real-time evaluation of the system’s integrity. Appropriate flow of contrast from the reservoir to the tubing and into the ventricles was easily visualized fluoroscopically. Digital storage of the real-time fluoroscopy images allowed for repeated review of the contrast injection to ensure that subtle areas of contrast extravasation outside the reservoir, tubing, or ventricles were not overlooked. The sensitivity of the examination was increased further by performing a flat panel CT scan after contrast was clearly identified extending throughout the ventricles. The data from this three-dimensional scan was reformatted to clearly demonstrate contrast confined to the Ommaya system and ventricles. Furthermore, the extra-axial collection overlying the right frontal lobe was shown to be free of contrast with excellent anatomic correlation to the prior non-invasive imaging. This study nicely demonstrated the integrity of the Ommaya system and affected the clinical decision to avoid Ommaya reservoir replacement at the time of subdural collection evacuation.

Although not performed in this case report, real time fluoroscopic evaluation of Ommaya reservoir integrity may be best performed in hybrid neurointerventional-surgical suites. These modern suites offer state of the art fluoroscopic equipment in an operative suite that would allow for evaluation of the Ommaya reservoir integrity as described in this report followed by the neurosurgical intervention that is tailored to the imaging findings. This streamlined approach would save time and resources by reducing patient transfer times, the need for repeat anesthesia set up, and repeat procedural room turn around. It will be of interest to see how similar diagnostic questions may be answered in an innovative manner using these hybrid suites. 

## Conclusions

Real-time fluoroscopic and flat panel CT evaluation of Ommaya reservoir integrity by iodinated contrast injection may be safely performed to evaluate the integrity of the catheter system. Demonstration of Ommaya system integrity without evidence of blockage or leakage in this case report allowed for tailored neurosurgical treatment. 
